# Association between frailty and meaning in life of older adults in nursing home: the mediating effect of psychological resilience

**DOI:** 10.3389/fpsyg.2024.1365817

**Published:** 2024-06-17

**Authors:** Jiquan Zhang, Fan Xu, Yang Zhou, Jijun Wu, Yuxin Li, Wei Qing

**Affiliations:** ^1^Department of Nursing, Deyang People's Hospital, Deyang, China; ^2^Department of Science and Education, Deyang People's Hospital, Deyang, China

**Keywords:** older adults, meaning in life, frailty, psychological resilience, mediating effect

## Abstract

**Background:**

Meaning in life is a crucial aspect of psychological well-being, often overlooked despite its clinical significance. This warrants further investigation, especially regarding its relationship with frailty and psychological resilience.

**Objective:**

This study aims to assess the status and relevance of frailty, psychological resilience, and meaning in life among older adults in Chinese nursing homes. Additionally, it explores the mediating role of psychological resilience between frailty and meaning in life, providing insights to improve the meaning in life for older adults in nursing homes.

**Methods:**

Between August 2022 and November 2022, 302 older adults in Chinese nursing homes were selected using convenience sampling. The study utilized the Socio-demographic Characteristics Questionnaire, Tilburg Frailty Indicator, Connor-Davidson Resilience Scale, and the Source of Meaning Scale for Older Adults. A face-to-face questionnaire survey was conducted, and SPSS 27.0 was employed for analyzing correlations between frailty, psychological resilience, and meaning in life. The mediating effect of psychological resilience was assessed using Model 4 in the Process plug-in.

**Results:**

Older adults in nursing homes exhibited a frailty total score of 4.00 (2.00, 5.00), with a prevalence of 28.5%. Psychological resilience scored 66.00 (51.75, 76.00), and meaning in life scored 149.00 (132.00, 158.25). Frailty showed a negative correlation with both meaning in life and psychological resilience, while meaning in life demonstrated a positive correlation with psychological resilience. Psychological resilience exhibited a partial mediating effect, accounting for 51.04% of the total effect between frailty and meaning in life.

**Conclusion:**

Frailty incidence is high among older adults in nursing homes, with psychological resilience at a general level and meaning in life in the upper middle level. Psychological resilience plays a crucial role as a partial mediator between frailty and meaning in life. Timely assessment of frailty, targeted interventions, and improvements in psychological resilience are essential for enhancing the meaning in life and promoting successful aging.

## 1 Introduction

Population aging is a significant demographic trend globally and within China, with recent years showing a growing trend (Fang et al., 2020). According to the “2021 National Aging Development Bulletin,” individuals aged 60 and above constitute 18.7% of China’s total population, with 191 million people aged 65 and over, accounting for 13.5%. By approximately 2035, China’s adults aged 60 and above will exceed 400 million, marking the country’s entry into an aging society stage. As the population has aged and family situations have changed in China, by the end of 2021, there were 358,000 nursing homes with 8.159 million available beds (Zhou et al., 2023). As part of China’s long-term care system, nursing homes play a significant role in healthy aging. However, due to the late development of the nursing home system in China, most facilities face challenges such as a lack of professional nursing staff and the need for improvements in security services (Chen et al., 2021). Understanding the determinants influencing high satisfaction with health status is essential in the context of older people’s health. Key factors include maintaining functional abilities, absence of physical and psychological illnesses, adequate physical activity levels, regular use of health and social services, and satisfaction with the quality of these services (Agustí et al., 2023). For older adults, significant aspects impacting quality of life are closely linked to health, interpersonal relationships, functional autonomy, and maintaining an active life. These elements are the main drivers determining the quality of life of older people, contrasting with economic considerations such as financial situation, pension, housing, or income (Parra-Rizo, 2017). According to E. H. Erikson’s psychological development theory, Zhang R. et al. (2017) and Zhang R. et al. (2017) found that meaning in life can provide energy support for older adults to complete the final task of life and promote successful aging. The stronger the meaning in life for older adults, the higher their cognition and satisfaction with their current social status, leading to higher quality of life.

With the introduction of the concept of successful aging and the advancement of positive psychology, meaning in life has garnered attention as a crucial factor in improving the subjective well-being and life satisfaction of older adults. Meaning in life denotes an individual’s perception and pursuit of the purpose and value of their existence, holding a pivotal role at the spiritual level (Zhou et al., 2019). It aids in mobilizing positive emotions, maintaining adaptive psychology and behavior, alleviating negative emotions such as loneliness, anxiety, and depression, and promoting mental health (Liu, 2017). Meaning in life is found in connection with self and others, with human relationships being the main source of meaning in life for older individuals (Hupkens et al., 2018). Aspects such as health, high socio-economic status, social relations, activities, and religion are associated with experiencing meaning in later life. Meaning in life and social support are important factors in promoting the successful aging of older adults in nursing homes (Xu et al., 2024). The social environment plays a crucial role in satisfying the need for meaning and can also be a cause for the loss of meaning. Apart from family ties, relationships with neighbors and professional caregivers are identified as sources of meaning (Duppen et al., 2019).

Frailty, refers to a complex clinical state characterized by acute changes in body health, decreased ability to maintain homeostasis, and reduced resistance to disease and stress due to age-related physiological and organ system declines (Lu et al., 2022). With ongoing research on the health of older adults, frailty has emerged as an important criterion for evaluating their health status, garnering sustained and widespread global concern (Clegg et al., 2013). Frailty not only limits the body’s ability to respond to internal and external stressors and maintain internal stability but also increases susceptibility to stressful events (Faria and Carmo, 2016). It elevates the risk of negative health outcomes such as falls, hospitalization, disability, and death, significantly reducing healthy life expectancy and quality of life in older adults (Vermeiren et al., 2016). The aging process is profoundly heterogeneous, with considerable variations in individuals’ lived experiences across physical, social, and psychological functioning domains throughout life (Cosco et al., 2017). Studies indicate a significant negative correlation between frailty and meaning in life among older adults (Golovchanova et al., 2021). Greater frailty is associated with a higher likelihood of feeling useless, leading to a diminished sense of meaning in life (Liu et al., 2018).

Moving into a nursing home entails leaving the original family environment and relatives and friends, placing older adults under pressure to adapt to their new surroundings (Faria and Carmo, 2016). Additionally, loneliness can diminish older adults’ participation in social activities and communication, increasing their sense of social isolation. Consequently, older adults in institutional settings often experience more psychological distress, such as depression, compared to those in the community or receiving home care (Zhang R. et al., 2017; Zhang X. et al., 2017). With a growing older population facing age-related challenges, the psychological resilience demonstrated by individuals has been associated with a reduced risk of depression, which is increasingly significant for older adults, caregivers, and clinicians (Kalomo et al., 2022). Psychological resilience, defined as the adaptive process in the face of adversity, sadness, threat, trauma, or other negative experiences (Fu and Wang, 2020), has been found to decrease with increasing frailty severity (Wang et al., 2020). Simultaneously, scholars have noted that higher levels of psychological resilience correspond to clearer life meanings and purposes (Hu et al., 2020).

Chinese scholars have relatively recently begun studying the meaning in life of older adults, and no research has yet reported on the relationship between the degree of frailty, psychological resilience, and meaning in life among older adults in nursing homes. This study aims to investigate the current status of frailty, psychological resilience, and meaning in life among older adults in nursing homes and to explore the mediating role of psychological resilience in the relationship between frailty and meaning in life, providing a theoretical basis for formulating measures to promote successful aging in this demographic.

The research hypotheses of this study included:

*H1*: Frailty is negatively correlated with psychological resilience and meaning in life.

*H2*: Psychological resilience is positively correlated with meaning in life.

*H3*: Psychological resilience mediates the relationship between frailty and meaning in life.

## 2 Methods

### 2.1 Participants

From August 2022 to November 2022, the convenience sampling method selected 302 permanent older adults from six nursing homes in China. Inclusion criteria were: (1) Age ≥ 60 years; (2) Residency in the care facility for ≥12 months; (3) Voluntary participation in the survey with informed consent obtained. Exclusion criteria included patients with cognitive impairment, consciousness disorders, language communication disorders, or other cooperation impediments. The sample size was determined using the cross-sectional survey sample size formula *n* = ${\left(u_{\alpha }\sigma /\delta \right)}^{2}$ . Taking the permissible error (δ) as 3.4 and the significance level (α) as 0.1, and based on previous literature indicating a standard deviation of 33.64 for the total score of meaning in life among older adults (Ma, 2022), the required sample size was calculated to be 265. Considering a 10% sample loss rate, the minimum sample size was estimated to be approximately 292 cases. A total of 310 questionnaires were distributed, and after eliminating those with evident patterns and inconsistent responses to homogeneous items, 302 valid questionnaires were recovered, yielding an effective recovery rate of 97.42%.

### 2.2 Procedures

A questionnaire survey method was employed to conduct one-to-one surveys with patients in nursing homes who met the inclusion and exclusion criteria. Firstly, the researchers obtained consent from the directors of the nursing homes. Secondly, the study’s purpose, significance, and precautions were explained to the participants. After obtaining informed consent, participants anonymously completed the questionnaires. A standardized instruction was provided during the survey, with on-the-spot assistance available for those facing difficulties. Data were collected by trained researchers using pen and paper. Trained researchers provided support to participants who requested help in reading the questionnaire. Participants took approximately 20 min to complete the questionnaires. After recovering the questionnaires, checks were conducted to ensure completeness and accuracy.

### 2.3 Measures

#### 2.3.1 Socio-demographic information

Socio-demographic information included gender, age, education level, marital status, number of children, family *per capita* monthly income, lifestyle factors (drinking, smoking, diet, exercise, sleep), number of chronic diseases, and self-rated health status.

#### 2.3.2 Chinese version of Tilburg frailty scale

The Tilburg Frailty Scale (Tilburg Frailty Indicator, TFI), compiled by Gobbens et al. (2010) from Tilburg University in the Netherlands, assesses self-rated frailty in older adults. The Cronbach’s α coefficient of the scale was 0.74. The Chinese version, translated by Xi et al. (2013), had a Cronbach’s α coefficient of 0.686. The scale comprises three dimensions: physical frailty (8 items), psychological frailty (4 items), and social frailty (3 items), totaling 15 items (e.g., “Have you felt depressed in the last month?”). Scores range from 0 to 15 points, with ≥5 points indicating frailty. The scale demonstrated a Cronbach’s α coefficient of 0.666 in this study.

#### 2.3.3 Chinese version of psychological resilience scale

The Connor-Davidson Resilience Scale 25 (CD-RISC 25), compiled by Connor and Davidson (2003), showed a Cronbach’s α coefficient of 0.74, evaluating an individual’s psychological resilience level. The Chinese version was revised by Yu et al. (2007), with a Cronbach’s α coefficient of 0.91. The scale comprises three dimensions: Toughness (13 items), strength (8 items), and optimism (4 items), totaling 25 items (e.g., “I do not give up easily when things do not look promising”), using a Likert 5-level scoring method (never, rarely, sometimes, often, and always were counted as 0 to 4 points). Scores range from 0 to 100 points, where higher scores indicate greater psychological resilience. A psychological resilience score < 60 points is considered poor, 60–69 points is general, 70–79 points is good, and ≥ 80 points is excellent. The Cronbach’s α coefficient of the scale in this study was 0.958.

#### 2.3.4 Meaningful sources of life scale for the older adults

Compiled by Zhou (2019), the Source of Meaning Scale for Older Adults evaluates the level of meaning in life experienced by older adults in China. The scale comprises 6 dimensions: family (4 items), sense of value (7 items), social support (4 items), leisure activities (5 items), personal development (4 items), and life security (4 items), totaling 28 items (e.g., “I can be trusted and respected by others”). It utilizes a Likert 7-point scoring method, where scores range from “completely meaningless” to “very much meaning,” recorded as 1–7 points. Scores range from 28 to 196 points, with higher score indicating a higher level of meaning in life. The scale demonstrated a Cronbach’s α coefficient of 0.924, and in this study, it exhibited a Cronbach’s α coefficient of 0.945.

#### 2.3.5 Statistical method

Statistical analysis utilized SPSS 27.0 software. Data distribution was assessed using a histogram and K-S test, revealing a skewed distribution. Count data were presented as *n* (%), while measurement data were described as M (P_25_, P_75_). Group comparisons were made using the non-parametric rank sum test. Spearman correlation analysis explored the correlation between frailty, psychological resilience, and meaning in life. The Harman single-factor test, using exploratory factor analysis, assessed common method deviation. Additionally, Model 4 in the Process plug-in analyzed the mediating effect of psychological resilience between frailty and meaning in life. The significance level was set at *α* = 0.05, and differences were deemed statistically significant at *p* < 0.05.

#### 2.3.6 Ethical considerations

This research adhered to the ethical standards outlined in the Declaration of Helsinki and received approval from the Deyang People’s Hospital Ethics Committee (approval number: 202204023 K01). Informed consent was obtained from all participants before their involvement. The survey ensured participant anonymity, and the confidentiality of information was guaranteed.

## 3 Results

### 3.1 Socio-demographic characteristics of the participants

Among the 302 respondents, 133 were male (44.0%) and 169 were female (56.0%). The median age was 68 (64, 73) years old, with males having a median age of 68 (65, 76) and females 67 (63, 72) years old. Education levels varied, with 60 cases (19.9%) being illiterate, 112 cases (37.1%) having primary school education, 93 cases (30.8%) completing junior high school, 18 cases (6.0%) finishing senior high school (including secondary school), and 19 cases (6.3%) holding junior college or higher qualifications. The majority, 265 cases (87.7%), were married. The number of children ranged from 1 to 2 in 230 cases (76.2%). Additionally, 165 cases (54.6%) reported a family *per capita* monthly income exceeding 5,000 yuan. Furthermore, 199 cases (65.9%) reported never consuming alcohol, and 230 cases (76.2%) either had no smoking habit or had quit smoking. A total of 242 cases (80.1%) adhered to a reasonable diet. Regular exercise was noted in 120 cases (39.7%), and 124 cases (41.1%) reported having normal sleep patterns. Additionally, 242 individuals (80.1%) among the older adults had 1 or 2 chronic diseases. Self-rated health status varied, with 35 cases (11.6%) reporting poor health, 118 cases (39.1%) describing it as general, 66 cases (21.9%) as good, 61 cases (20.1%) as very good, and 22 cases (7.3%) as excellent.

### 3.2 Comparison of the total score of the meaning in life of the older adults in nursing home with different characteristics

The non-parametric rank sum test was used to compare the total scores of meaning in life among older adults in nursing homes with different characteristics. Results indicated statistically significant differences in the total scores based on education levels, marital status, family *per capita* monthly income, exercise, sleep, number of chronic diseases, and self-rated health (*p* < 0.05). Refer to Table 1 for a detailed comparison of the total scores of meaning in life of older adults with different characteristics.

**Table 1:** Comparison of total scores of meaning in life of the older adults with different characteristics (*n* = 302).

Project	Item	*N* (%)	Meaning in life M (P_25_, P_75_)	*Z*	*P*
Gender	Male	133 (44.0)	149.00 (132.00, 161.00)	−0.981	0.327
	Female	169 (56.0)	149.00 (132.00, 158.00)		
Age	60-	174 (57.6)	149.00 (137.00, 160.00)	4.574	0.102
	70-	98 (32.5)	150.00 (129.00, 156.00)		
	80-	30 (9.9)	143.50 (118.00, 158.00)		
Level of education	Illiterate	60(19.9)	140.00 (122.00, 156.50)	19.823	0.001
	Primary school	112 (37.1)	145.50 (127.50, 157.00)		
	Junior high school	93 (30.8)	152.00 (140.00, 160.00)		
	High school	18 (6.0)	155.00 (149.00, 160.00)		
	University	19 (6.3)	158.00 (145.00, 175.00)		
Marital status	Married	265 (87.7)	150.00 (136.00, 159.00)	18.694	<0.001
	Be widowed	28 (9.3)	126.50 (109.50, 142.50)		
	Divorce	9 (3.0)	137.00 (128.00, 139.00)		
Number of children	0	4 (1.3)	134.00 (102.00, 146.00)	3.857	0.145
	1 ~ 2	227 (75.2)	149.00 (135.00, 159.00)		
	≥3	71 (23.5)	148.50 (120.00, 158.00)		
Family *per capita* monthly income	<2000	51 (16.9)	139.00 (117.00, 158.00)	20.408	<0.001
	2000–5,000	86 (28.5)	140.00 (126.00, 152.00)		
	>5,000	165 (54.6)	153.00 (142.00, 159.00)		
Drinking	Never	199 (65.9)	148.00 (132.00, 158.00)	0.982	0.612
	Sometimes	70(23.2)	150.00 (130.00, 159.00)		
	often	33 (10.9)	150.00 (145.00, 153.00)		
Smoking	Yes	230 (76.2)	149.50 (132.00, 159.00)	−1.228	0.219
	No	72(23.8)	146.50 (128.00, 158.00)		
Diet	Normal	242 (80.1)	149.00 (133.00, 158.00)	1.407	0.495
	Meat-based	39 (12.9)	152.00 (131.00, 165.00)		
	Vegetarian-based	21 (7.0)	148.00 (122.00, 158.00)		
Exercise	Very few	75(24.8)	136.00 (115.00, 155.00)	30.108	<0.001
	Sometimes	107 (35.4)	148.00 (132.00, 157.00)		
	often	120 (39.7)	153.00 (146.00, 161.50)		
Sleep	Normal	124 (41.1)	152.00 (137.00, 164.00)	13.246	0.001
	Sometimes insomnia	111 (36.8)	148.00 (135.50, 157.00)		
	Frequent insomnia	67 (22.2)	146.00 (118.00, 156.00)		
Number of chronic diseases	0	6 (2.0)	176.50 (167.00, 184.00)	13.301	0.001
	1 ~ 2	242 (80.1)	149.00 (132.00, 158.00)		
	≥3	54 (17.9)	148.00 (132.00, 158.00)		
Self-rated health status	Not good	35 (11.6)	137.00 (117.50, 155.50)	15.637	0.004
	General	118 (39.1)	146.00 (127.00, 158.00)		
	Good	66 (21.9)	150.00 (138.00, 162.00)		
	Better	61 (20.1)	152.00 (140.00, 160.00)		
	Very good	22 (7.3)	153.00 (141.00, 164.00)		

### 3.3 The total score of frailty, psychological resilience, meaning in life and the score of each dimension of the older adults in nursing homes

The survey results reveal that the total score of frailty among older adults in nursing homes is 4.00 (2.00, 5.00), the total score of psychological resilience is 66.00 (51.75, 76.00), and the total score of meaning in life is 149.00 (132.00, 158.25). Among the 302 older adults in nursing homes, the prevalence of frailty was 28.5% (86 cases), consisting of 38 males and 48 females. Detailed data, including the scores for each dimension, are presented in Table 2.

**Table 2:** The total score of frailty, psychological resilience, meaning in life, and the score of each dimension of older adults in nursing homes (*n* = 302).

Project	Item	Score M(P_25_, P_75_)	Item average score M(P_25_, P_75_)	Min	Max
Body frailty	8	2.00 (1.00, 3.00)	0.25 (0.13, 0.38)	0	8
Psychological frailty	4	1.00 (1.00, 2.00)	0.25 (0.25, 0.50)	0	4
Social frailty	3	1.00 (0.00, 1.00)	0.33 (0.33, 0.33)	0	3
Total frailty score	15	4.00 (2.00, 5.00)	0.27 (0.13, 0.33)	0	15
Toughness	13	33.00 (26.00, 39.00)	2.54 (2.00, 3.00)	0	52
Power	8	22.00 (16.75, 24.00)	2.75 (2.09, 4.00)	5	32
Optimistic	4	10.00 (8.00, 13.00)	2.50 (2.00, 3.25)	2	16
Total score of psychological resilience	25	66.00 (51.75, 76.00)	2.64 (2.07, 3.04)	11	98
Family	4	21.00 (19.00, 23.25)	5.25 (4.75, 5.81)	8	28
Social support	4	22.00 (19.00, 24.00)	5.50 (4.75, 6.00)	5	28
Sense of value	7	37.00 (33.00, 41.00)	5.29 (4.71, 5.86)	7	49
Leisure activities	5	27.00 (22.00, 29.00)	5.40 (4.40, 5.80)	5	35
Personal development	4	20.00 (14.75, 22.00)	5.00 (3.69, 5.50)	4	28
Life security	4	22.00 (20.00, 25.00)	5.50 (5.00, 6.25)	7	28
The total score of life meaning	28	149.00 (132.00, 158.25)	5.32 (5.71, 5.65)	37	196

### 3.4 Correlation analysis of frailty, psychological resilience and total score of meaning in life of the older adults in nursing homes

Spearman correlation analysis revealed significant associations among frailty, psychological resilience, and the total score of meaning in life in older adults residing in nursing homes. The degree of frailty demonstrated a negative correlation with the total score of psychological resilience (rs = −0.376, *p* < 0.001) and a negative correlation with the total score of meaning in life (rs = −0.337, *p* < 0.001). Furthermore, the total score of psychological resilience exhibited a positive correlation with the total score of meaning in life (rs = 0.523, *p* < 0.001). Refer to Table 3 for a comprehensive presentation of the correlation analysis.

**Table 3:** Correlation analysis of frailty, psychological resilience, and total score of meaning in life of older adults in nursing homes (*n* = 302).

Project	Frailty	Psychological resilience	Meaning in life
Frailty	1.000		
Psychological resilience	−0.376^**^	1.000	
Meaning in life	−0.337^**^	0.523^**^	1.000
Family	−0.208^**^	0.319^**^	_
Social support	−0.294^**^	0.533^**^	_
Sense of value	−0.370^**^	0.558^**^	_
Leisure activities	−0.259^**^	0.409^**^	_
Personal development	−0.260^**^	0.354^**^	_
Life security	−0.122^*^	0.175^**^	_

^*^Means *P* < 0.05, ^**^means *P* < 0.01.

### 3.5 Analysis of the mediating effect of psychological resilience between frailty and meaning in life

**Figure 1:**
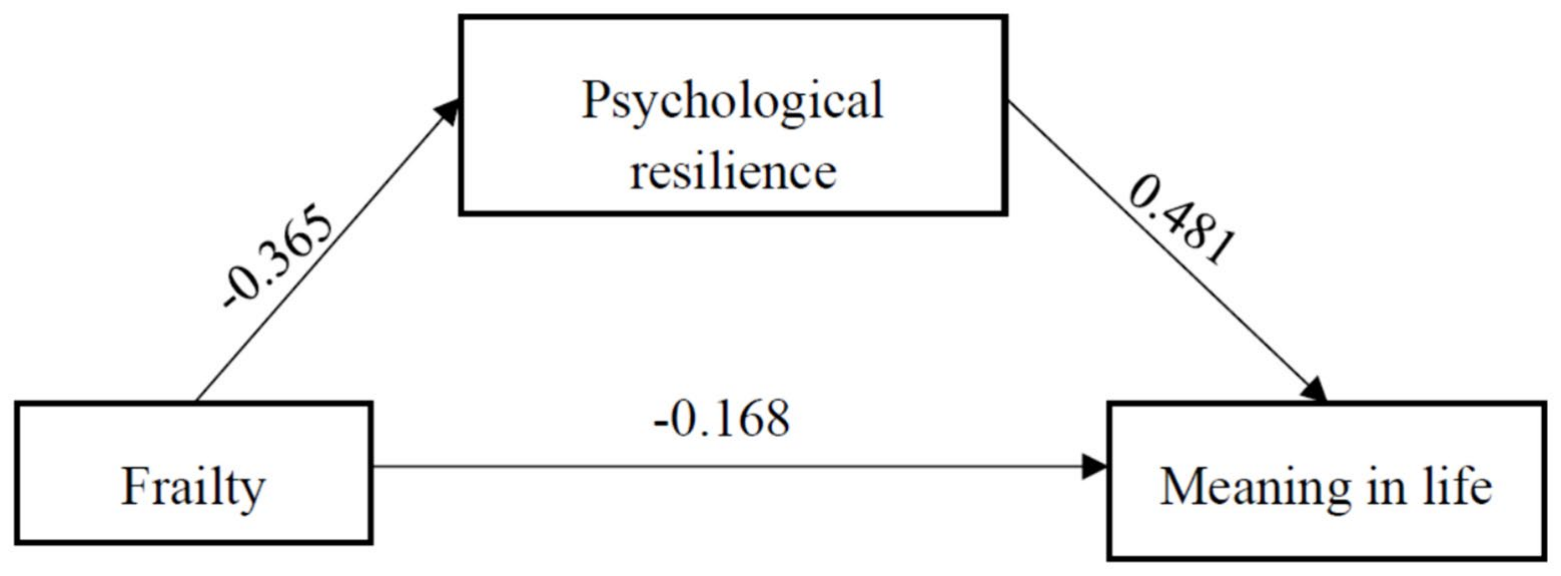
Mediating effect model of psychological resilience between frailty and meaning in life (non-standardized).

The Harman single-factor test method was employed for exploratory factor analysis to examine common method bias. Results revealed that the unrotated first factor accounted for 30.31% of the variance, which is below the critical value of 40%, indicating an absence of substantial common method bias in this study (Tang and Wen, 2020). Following the standardization of all variables, Model 4 within the Process plug-in was used to analyze the mediating role of psychological resilience between the degree of frailty and meaning in life. The bias-corrected bootstrap confidence interval estimation method, involving 5,000 iterations, was applied to assess the significance of the mediating effect, establishing a 95% confidence interval. Outcomes indicated a significant negative predictive effect of frailty on psychological resilience (*β* = −0.365, *t* = −6.799, *p* < 0.001), a significant negative predictive effect on meaning in life (*β* = −0.168, *t* = −3.285, *p* = 0.001), and a significant positive predictive effect on meaning in life (*β* = 0.481, *t* = 9.381, *p* < 0.001). The 95% confidence interval of the direct effect of frailty on meaning in life and the mediating effect of psychological resilience does not contain 0. This signifies that psychological resilience exerts a partial mediating effect between frailty and meaning in life. The mediating effect of psychological resilience is a * b = −0.365 × 0.481 = −0.176. The direct effect of frailty on meaning in life of older adults in nursing homes is −0.168, the total effect of frailty on meaning in life is −0.344, and the contribution rate of mediating effect to the total effect is Effect *M* = 0.176/0.344 = 51.04%. Refer to Figure 1 and Table 4 for a detailed display of the mediating effect model.

**Table 4:** The mediating effect of psychological resilience between frailty and meaning in life of older adults in nursing homes (non-standardized).

Item	*β*	*P*	95%CI
Total effect	−0.344	<0.001	−0.451, −0.237
Direct effect	−0.168	0.001	−0.269, −0.068
Indirect effect	−0.176	–	−0.266, −0.103

## 4 Discussion

Frailty poses a significant challenge in the aging process, impacting not only the quality of life for older adults but also imposing additional financial strain on families and affecting the overall harmony and health of society. The findings of this study revealed a frailty incidence of 28.5% among older adults in nursing homes, closely consistent with the research of Liu et al. (2022), indicating a high prevalence of frailty in such settings at 30.11%. Gender disparities were observed, with studies suggesting a higher prevalence of frailty in older adult women compared to men (Park and Ko, 2021). In this study, where the majority of nursing home residents were women, the prevalence might be influenced by factors such as declining estrogen levels post-menopause, leading to health-related challenges such as lack of vitamin D, accelerated loss of calcium, and significant degradation of bone, muscle, and other functions (Zhao and Yan, 2022). Additionally, lower education levels and a higher prevalence of chronic diseases in this population could contribute to the elevated frailty detection rate (Hanlon et al., 2018; Chen and Shen, 2022; Wang et al., 2022). In this study, most older adults in the old-age care institutions were not highly educated, and 80.1% of them had 1 to 2 chronic diseases. These factors may lead to a higher detection rate of frailty among older adults in old-age care institutions in this study.

Psychological resilience, crucial for adaptive responses to adversity and trauma, emerged in this study as being at a general level among older adults in nursing homes, reflecting individuals’ mental health. The study findings reveal that the psychological resilience of older adults in nursing homes aligns with similar research conducted in China (Han et al., 2021). It is noted that a favorable economic status acts as a protective factor in maintaining the mental well-being of older individuals (Zhang and Yang, 2017). Mental resilience levels were reported to be higher among older adults who are male, in good health, and without chronic diseases (Majnarić et al., 2021; Rodrigues and Tavares, 2021). However, in the context of this study, the majority of older adults in the nursing home are women. Additionally, 46.4% of the older adults in the nursing home have a *per capita* monthly income of ≤5,000 yuan, 11.6% have poor self-perceived health status, 39.1% have general self-perceived health status, and up to 80.1% have 1 or 2 chronic diseases. These factors may contribute to the low level of psychological resilience among older adults in the nursing home in this study.

Erickson’s theory of psychosocial development suggests that in the older adult stage, individuals often reflect on their lives and ponder their life’s value. Those with a positive attitude adapt and enhance themselves through introspection, overcoming the decline in physical function, and finding happiness and serenity in life’s meaning. The total score for meaning in life among older adults in nursing homes in this study was 149.00 (132.00, 158.25), exceeding the median score of 98.00. These results align with (Liang et al.’s, 2021) findings. Protective factors such as higher education level, being married, higher economic status, absence of chronic disease, good physical function, and regular exercise contribute to the meaning in life for older individuals (Jiang et al., 2018; Wang, 2021; Chen et al., 2022). Conversely, risk factors such as smoking are linked to lower meaning in life (Guo et al., 2022). Moreover, older adults who rated their health status higher reported a higher level of life meaning. Notably, within this study, most older adults in old-age care institutions were married. Additionally, a significant proportion of them maintained a healthy lifestyle, avoiding habits like smoking and drinking while actively participating in regular exercise. These factors collectively suggest a potential association with the elevated levels of life meaning observed among the older adults in the old-age care institutions in this study.

The Spearman correlation analysis findings revealed a significant negative correlation between the degree of frailty among older adults in nursing homes and their perceived meaning in life (*p* < 0.01). Subsequent mediating effect analysis indicated a substantial predictive effect on meaning in life (*β* = −0.168, *t* = −3.285, *p* = 0.001). This result underscores that as the degree of frailty of the older adults in nursing homes increases, their level of meaning in life diminishes. Conversely, a milder degree of frailty corresponds to a higher level of meaning in life. Frailty in older adults is a syndrome closely related to their physical and mental health, predominantly manifesting in the deterioration of physical function, especially the loss of mobility, which seriously affects their self-care ability and quality of life. Studies have pointed out that the decline in physical function affects older adults’ views of life and attitudes towards death (Haugan, 2014). In particular, those with low self-care abilities often become reliant on others for assistance, leading them to perceive themselves as burdens to their children and families, fostering a sense of uselessness, life meaninglessness, and a diminished overall meaning in life. Older adults with less frailty have relatively healthy and stable physical, psychological, and social relationships, have more energy to take care of their families and participate in social activities, and consequently experience a richer meaning in life.

The investigation findings indicate that resilience partially mediates the correlation between frailty and meaning in life among older adults in nursing homes. This mediating effect amounts to 51.04% of the total effect. The frailty levels of older adults not only directly influence the perception of meaning in life but also indirectly impact it through psychological resilience. Existing research has shown that increased stressors, decreased functional capabilities, social isolation, loneliness, and chronic health conditions can detrimentally affect the mental well-being of older individuals (Aydın et al., 2020). When faced with incapacitating challenges, older adults continually adjust to accommodate declines in physical, psychological, and social aspects. This process may result in diminished self-confidence and a sense of control, hindering the effective mobilization of psychosocial resources necessary for maintaining mental health equilibrium and thereby reducing psychological resilience. The resilience integration model posits that the emergence of stress and adversity enables individuals originally in a “physical and mental balance” to mobilize protective factors to maintain equilibrium between themselves and the environment. However, if the pressure is excessive and resistance proves ineffective, this equilibrium collapses (Ma et al., 2008). Older adults with a low level of resilience are more prone to feelings of hopelessness and frustration in life because they struggle to rationally cope with setbacks and pressures, resulting in a lower level of meaning in life. Conversely, those with lesser frailty can efficiently mobilize both internal and external resources when confronting adversity, resulting in higher levels of psychological resilience. During such periods, older adults tend to reinterpret past, present, and future life experiences with a positive and optimistic outlook, fostering a healthy life orientation and often experiencing heightened meaning in life. This underscores the vital psychological buffering role played by resilience between frailty and meaning in the lives of older adults in nursing homes.

The degree of frailty not only directly predicted a negative impact on meaning in life but also indirectly affected it through psychological resilience. Considering frailty’s influence on the meaning in life of older adults in nursing homes, strategies for prevention and intervention can be developed from both physiological and psychological perspectives to enhance their sense of meaning and promote healthy aging. Firstly, leveraging the potential reversibility of frailty, initiatives for disseminating health knowledge can foster a health-promoting mindset among older adults in care facilities. Additionally, encouraging their active engagement in exercise, making rational adjustments to dietary habits, cultivating a balanced lifestyle, and promoting physical well-being collectively contribute to reducing frailty levels. Secondly, providing professional psychological services, including mental health education, group counseling, and individual therapy, can enhance older adults’ coping skills in dealing with adversity, stress, or negative events. This, in turn, boosts their psychological resilience, maintaining their mental and emotional well-being, fostering healthy aging, and improving their overall quality of life.

### 4.1 Limitations

It’s crucial to acknowledge certain limitations in this study. Due to constraints in human, material, and financial resources, a cross-sectional survey was employed. Firstly, the findings only offer a snapshot of the situation at that specific point in time, and establishing the temporal sequence among variables is not feasible. Therefore, no inference can be drawn about causality. Secondly, it’s important to note that the sample in this study consisted solely of older adults from six nursing homes in a single province in China. The study did not undertake a large-scale investigation, thus the representativeness of the sample is somewhat limited. Future research could validate the conclusions drawn here through investigations across diverse regions, varied demographic strata, and larger sample sizes.

## Data availability statement

The original contributions presented in the study are included in the article/Supplementary materials, further inquiries can be directed to the corresponding authors.

## Ethics statement

The study approved by the Deyang People’s Hospital, Sichuan, China. The studies were conducted in accordance with the local legislation and institutional requirements. The participants provided their written informed consent to participate in this study.

## Author contributions

JZ: Data curation, Formal analysis, Methodology, Software, Validation, Writing – original draft, Writing – review & editing. FX: Data curation, Formal analysis, Methodology, Software, Supervision, Validation, Writing – original draft, Writing – review & editing. YZ: Data curation, Formal analysis, Investigation, Software, Writing – original draft. JW: Data curation, Formal analysis, Investigation, Writing – original draft. YL: Data curation, Formal analysis, Investigation, Writing – review & editing. WQ: Project administration, Resources, Supervision, Writing – review & editing, Writing – original draft.
